# Enterovirus Migration Patterns between France and Tunisia

**DOI:** 10.1371/journal.pone.0145674

**Published:** 2015-12-28

**Authors:** Ines Othman, Audrey Mirand, Ichrak Slama, Maha Mastouri, Hélène Peigue-Lafeuille, Mahjoub Aouni, Jean-Luc Bailly

**Affiliations:** 1 University of Monastir, Faculty of Pharmacy, LR99-ES27, Monastir, Tunisia; 2 University of Carthage, Faculty of Sciences of Bizerte, Tunisia; 3 Université d’Auvergne, EPIE, EA 4843, Clermont-Ferrand, France; 4 CHU Clermont-Ferrand, Service de Virologie, Centre National de Référence des Enterovirus–Parechovirus, Clermont-Ferrand, France; 5 Fattouma Bourguiba University Hospital, Laboratory of Microbiology, Monastir, Tunisia; University of Hong Kong, HONG KONG

## Abstract

The enterovirus (EV) types echovirus (E-) 5, E-9, and E-18, and coxsackievirus (CV-) A9 are infrequently reported in human diseases and their epidemiologic features are poorly defined. Virus transmission patterns between countries have been estimated with phylogenetic data derived from the 1D/VP1 and 3CD gene sequences of a sample of 74 strains obtained in France (2000–2012) and Tunisia (2011–2013) and from the publicly available sequences. The EV types (E-5, E-9, and E-18) exhibited a lower worldwide genetic diversity (respective number of genogroups: 4, 5, and 3) in comparison to CV-A9 (n = 10). The phylogenetic trees estimated with both 1D/VP1 and 3CD sequence data showed variations in the number of co-circulating lineages over the last 20 years among the four EV types. Despite the low number of genogroups in E-18, the virus exhibited the highest number of recombinant 3CD lineages (n = 10) versus 4 (E-5) to 8 (E-9). The phylogenies provided evidence of multiple transportation events between France and Tunisia involving E-5, E-9, E-18, and CV-A9 strains. Virus spread events between France and 17 other countries in five continents had high probabilities of occurrence as those between Tunisia and two European countries other than France. All transportation events were supported by BF values > 10. Inferring the source of virus transmission from phylogenetic data may provide insights into the patterns of sporadic and epidemic diseases caused by EVs.

## Introduction

Human enteroviruses (EVs) belong to a large genus within the *Picornaviridae* family (a group of non-enveloped viruses with a positive RNA genome) and they comprise genetically and antigenically distinct (sero/geno)types [**1]**. Human EV infections are most frequently mild and asymptomatic but acute infections are also reported sporadically or epidemically. Molecular methods have been increasingly used since the 1990s for the rapid diagnosis of EV infections and typing of EV strains in different clinical specimens. These methods were instrumental in accurately defining the epidemiological patterns of individual EV types and genogroups (or subtypes), and the changes over time and across geographic regions. A major example of such changes is the sustained increase in the prevalence of hand, foot and mouth disease (HFMD) caused by a large array of EV-A71 genogroups in countries of South Eastern Asia since the mid-1990s, during which time reports in Europe and the USA were mainly of sporadic infections [**2–4**]. A second example involving close relatives of EV-A71 is the recent increase in the reported number of HFMD outbreaks caused by coxsackievirus (CV)-A6 in Europe and Asia [**5–9**]. Thirdly, in 2014, two health alerts focused attention on an outbreak of severe respiratory infections in children in Colorado, USA, caused by a disregarded EV type designated EV-D68 [**10**]. Sporadic cases were reported in European countries [**11–13**]. The epidemiologic factors involved in these geographic and temporal variations are multifactorial and still incompletely understood.

The EV epidemic patterns at global and local geographic scales are mainly determined by herd immunity against each EV type. Other non-selective factors such as random transportation events of virus strains between countries by infected individuals may also be involved but have not been analyzed. Statistical phylogenetic methods developed recently have been used to address this question and applied to investigate virus migration of several virus groups other than EVs [**14–18**]. In an earlier study, we showed that virus transportation events were involved in the sporadic dissemination of EV-A71 between European countries in the 2000s [**19**].

To investigate this question further, the present collaborative study focused on two countries, France and Tunisia, and four EV types, echovirus 5 (E-5), E-9, and E-18, and CV-A9. The four EV types were usually associated with mild febrile infections and exanthemas [**20,21**]. They were reported in acute neurological manifestations, of which acute meningitis was the most frequent [**22,23**], while meningo-encephalitis, encephalitis, and flaccid paralysis were occasional [**24,25**]. Severe infections were reported in both immunocompetent and immunocompromised individuals [**26, 27**]. CV-A9 had an endemic pattern of circulation in the USA between 1970 and 2005, while the other three types displayed epidemic features [**28**]. E-18 and CV-A9 were involved in recent outbreaks of acute meningitis in Asia [**29,30**]. In France and Tunisia, sporadic detection of the four EV types has been reported [**31–35**]. Previous molecular epidemiology studies involved only E-9 and CV-A9 but did not address the issue of inferring their geographic spread from phylogenetic data [**36,37**].

The aim of the study was to investigate virus transportation patterns between France and Tunisia as estimated by phylogeographic analyses of two viral gene sequences (1D/VP1 and 3CD) and also to explore virus spread between other countries by combining the sequences determined with publicly available sequences.

## Materials and Methods

### Virus sample and ethics statement

The virus sample consisted of EV strains identified in clinical specimens (cerebrospinal fluid, throat swabs, and feces) obtained during routine virologic diagnosis in patients admitted to the hospitals of Clermont-Ferrand (France; 2000–2012; n = 58 strains) and Monastir (Tunisia; 2011–2013; n = 16) (**S1 Table**). The sample comprised 74 strains (E-5, n = 14; E-9, n = 16; E-18, n = 30; CV-A9, n = 14). A sample of 7 E-6 strains was also included to analyze the recombination patterns (see below). The study was performed with the leftover material of specimens collected for diagnostic purposes during hospitalization of patients. The specimens were used after de-identification of clinical data and analyzed anonymously. The study and use of stored samples for testing were approved by the local committee for the protection of human subjects (Comité d’Ethique des Centres d’Investigation Clinique de l’Inter-Région Rhône-Alpes-Auvergne, France) with a waiver of informed consent (institutional review board number 5044) and by the local Tunisian Ethic and Research Committee (CHU Fattouma Bourguiba, Monastir; Committee’s advice on 26 June 2014).

### Gene amplification of viral sequences and nucleotide sequencing

The 1D/VP1 and 3CD viral sequences were determined with previously described methods [**32,38**]. The 1D/VP1 sequences of 24 virus isolates in the sample have been reported previously (see **S1 Table**). The nt sequences determined in this study (1D/VP1, n = 50 sequences; 3CD, n = 78) were submitted to the European Molecular Biology Laboratory database (accession numbers LN876128–LN876255).

### E-5, E-9, E-18, and CV-A9 sequence data sets

The sequences determined were compared with 193 EV sequences available in public databases (**S2 Table**). The 1D/VP1 sequence datasets were constructed by collating all entries (as of May 2015; n = 162) including the nt sequence of the VP1 capsid protein for any isolate of human E-5, E-9, E-18, and CV-A9. Only entries reporting nt sequences with fully specified sampling dates and countries of origin were used, and those reporting sequences lower than 750 nt were discarded to conserve complete or near-complete gene sequences. The 1D/VP1 sequence datasets of each EV type were analyzed separately. The features of the sequence samples are summarized in **S3 Table** (datasets 1). Larger sequence samples (datasets 2) were constructed with 1D/VP1 gene sequences less than 500 nt in length to investigate whether the phylogeographic data inferred from the first datasets could be biased by their small size. The partial sequences selected for the second datasets were of different length (from 249 to 357 nt) and encompassed different nt positions within the 5’ part of the 1D/VP1 gene, depending on the EV type. All the second datasets included the full sets of complete (near-complete) sequences. The 3CD sequences were compared with 31 publicly available sequences.

### Phylogeographic analyses of sequence data

Genetic distances and neighbor joining trees were determined with the computer program MEGA version 5 [**39**]. The reliability of branching patterns was determined by the bootstrap resampling test with 1000 replicates [**40**]. The genealogical trees were reconstructed with the BEAST v1.8.2 program [**41**]. The phylogenies were inferred with the SRD06 substitution model [**42**], an uncorrelated lognormal relaxed clock model [**43**], and the Bayesian skyline plot model [**44**]. The values of all phylogenetic parameters were calculated with a Markov Chain Monte Carlo (MCMC) process involving 40−80 × 10^6^ generations to ensure convergence of parameter estimates. The Tracer v.1.5 program (http://evolve.zoo.ox.ac.uk/software) was used to check for convergence. The trees inferred during the MCMC process were sampled to obtain a final set of 10000 trees. Maximum clade credibility (MCC) trees were calculated with the TreeAnnotator v.1.8 program (http://evolve.zoo.ox.ac.uk/software) and topological support was assessed by estimating the values of the posterior probability (pp) density of each node.

A discrete phylogeographic model was used to analyze virus spread between countries [**15**]. The country of virus sampling was checked for each sequence to infer the geographic locations of nodes within the 1D/VP1 genealogies. A phylogenetic pattern indicative of a probable virus transportation event between two nodes was defined as follows: (1) the nodes exhibited pp>0.9, (2) the probabilities of inferred locations (p) were >0.5 at both nodes, and (3) the difference between the times of the most recent common ancestor (TMRCA) or the 95% highest posterior density (HPD) intervals estimated at the nodes were in a range of one year. This empirical interval was chosen because the dates of viral sequences were only defined by the sampling year whereas the month and day of virus isolation for publicly available sequences are commonly missing. The virus transportation events assessed over this short time interval were considered epidemiologically more relevant than those estimated over larger temporal intervals. The spatial diffusion history of each EV type was reconstructed by the phylogenetic analyses of complete (near-complete) and partial sequence datasets. A Bayesian stochastic search variable selection (BSSVS) approach was used to find a parsimonious set of rates explaining the migrations between discrete geographic locations in the phylogenies [**14]**. The phylogenetic data were analyzed with the Bayes factor (BF) test implemented in the SPREAD v1.0.4 program to determine the most probable virus migration events [**45**].

### Recombination analyses of VP1 and 3CD sequence data

The 1D/VP1 and 3CD sequence datasets were analyzed with the RDP computer program [**46**] to detect recombination events. The analysis provided statistical evidence that only one 3CD sequence (namely HF948106_E18_CF1442_FRA02) was recombinant. The 1D/VP1 and 3CD loci are 2260 nt apart (nt positions 2445–3305 and 5565–6461, respectively). Positions are given relative to the genome of the E-18/Metcalf strain (accession number AF317694). The occurrence of recombination between the two loci was investigated by analyzing partitioning of lineages in the 1D/VP1 and 3CD phylogenetic trees. On the basis of a previous study [**47**], a phylogenetic pattern indicated a recombination event between two distinct VP1 lineages when they clustered into a single 3CD lineage. When the 1D/VP1 lineages were from two EV types, the recombination was intertypic; otherwise, the recombination was intratypic. The recent recombination events were identified with the following additional conditions: (1) the 3CD sequences had >95% nt similarity. This threshold was chosen because it is generally accepted that the evolutionary rate of EV genomes is 1% per year and the molecular footprints of recombination events are rarely conserved more than five years. (2) the common ancestral node exhibited pp>0.9, and (3) the 95% HPD intervals of TMRCA values estimated for nodes that grouped equivalent clades in the 3CD and VP1 trees should overlap to infer probable intratypic recombination events. When the above criteria were not met, partitioning of lineages was considered to reflect an ancient, most probably intertypic, recombination.

## Results

### Clustering of E-5, E-9, E-18, and CV-A9 1D/VP1 sequences from France and Tunisia

**Fig 1:**
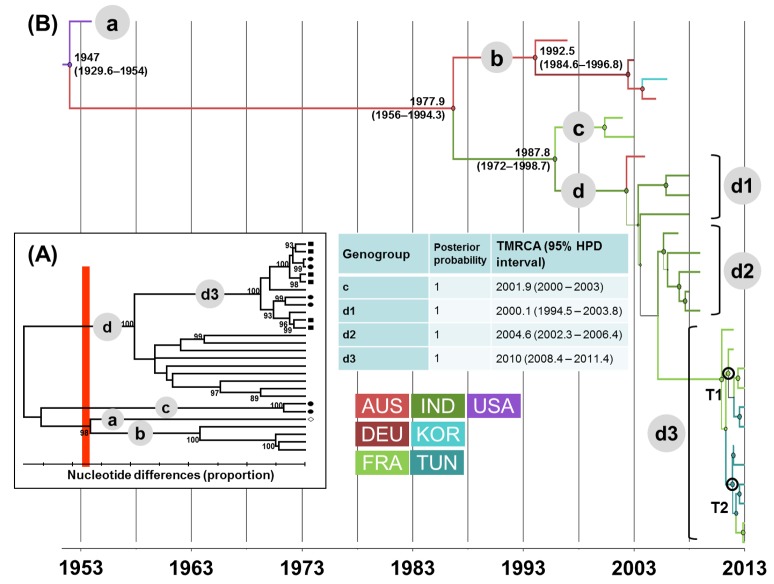
Genetic diversity and phylogenetic patterns of temporal and spatial distribution of echovirus 5. (A) The phylogenetic tree was estimated with the complete VP1 gene sequences and used to identify genetic clusters among the E-5 sequences. Only bootstrap values of greater than 70% are shown. The genogroups were differentiated at a divergence threshold of 15% nt differences (red bar) and designated by lower case letters in grey circles. (B) The maximum clade credibility tree was inferred with a Bayesian Markov chain Monte Carlo analysis and a discrete phylogeographic model. The tree branches are colored according to the geographic location estimated with the highest probability; the color code for geographic locations is indicated in the figure. The tree nodes are colored according to the most probable geographic location and the circle size is proportional to posterior probability (pp). A node shown with an open dark circle (and letter T followed by a number) indicates transportation of a virus strain between two countries. The time to the most recent common ancestor (TMRCA) and the highest posterior probability density (HPD) intervals are indicated for the main nodes in the tree and the table. The scale at the bottom of the figure indicates calendar years. Abbreviations used: AUS, Australia; DEU, Germany; FRA, France; IND, India; KOR, Korea; TUN, Tunisia; USA, United States of America.

**Fig 2:**
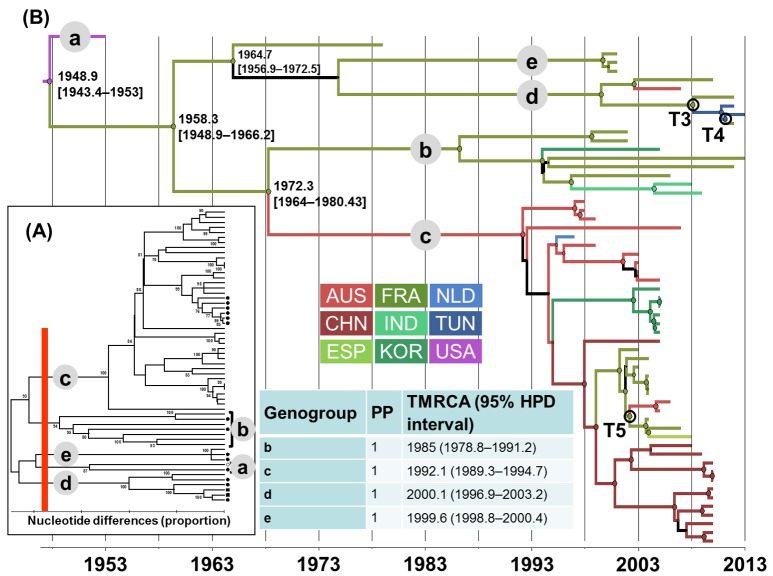
Genetic diversity and phylogenetic patterns of temporal and spatial distribution of echovirus 9. See the legend to Fig 1 for detailed description. Abbreviations used: AUS, Australia; CHN, China; ESP, Spain; FRA, France; IND, India; KOR, Korea; NLD, Netherlands; TUN, Tunisia; USA, United states of America.

**Fig 3:**
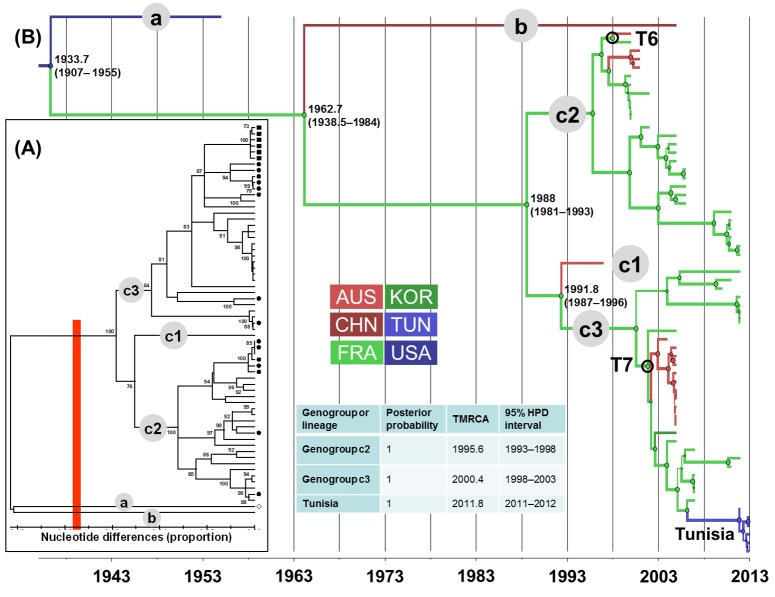
Genetic diversity and phylogenetic patterns of temporal and spatial distribution of echovirus 18. See the legend to Fig 1 for detailed description. Abbreviations used: AUS, Australia; CHN, China; FRA, France; KOR, Korea; TUN, Tunisia; USA, United States of America.

**Fig 4:**
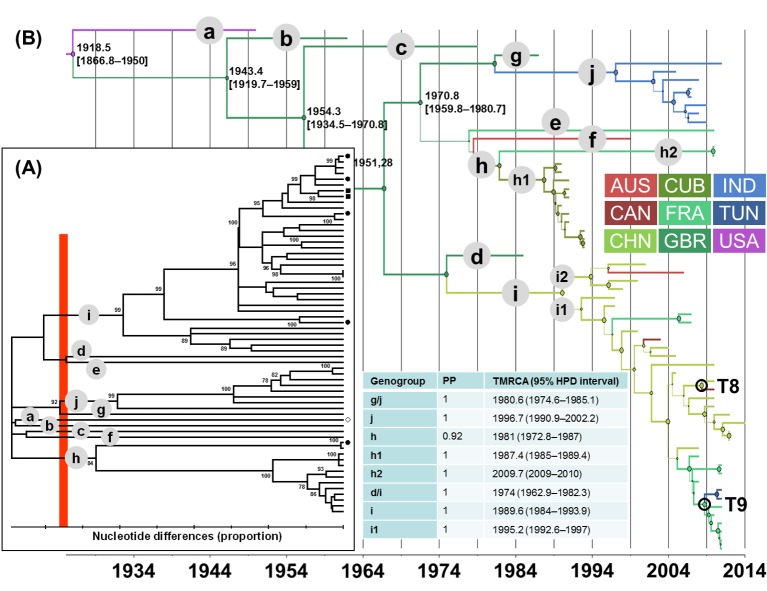
Genetic diversity and phylogenetic patterns of temporal and spatial distribution of coxsackievirus A9. See the legend to Fig 1 for detailed description. Abbreviations used: AUS, Australia; CAN, Canada; CHN, China; CUB, Cuba; FRA, France; GBR, Great Britain; IND, India; TUN, Tunisia; USA, United States of America.

In the phylogenetic trees shown in Figs 1A, 2A, 3A and 4A, the EV lineages were allocated to different genogroups on the basis of a threshold of 15% nt differences [48]. The genogroups were designated sequentially by letters according to the TMRCA values collated from the MCC trees inferred with the 1D/VP1 sequence datasets (Figs 1B, 2B, 3B and 4B). The overall E-5, E-9, E-18, and CV-A9 lineages were thus assigned to 4, 5, 3, and 10 genogroups, respectively. The sequences sampled in France or Tunisia were assigned to lineages that displayed consistent bootstrap values of 100%. The E-5 sequences of both countries clustered within 2/4 genogroups (c and d) and the E-9 sequences within 4/5 genogroups (b to e). The E-18 sequences were assigned to two lineages within the same genogroup c and those of CV-A9 to genogroups h and i. All the sequences sampled in Tunisia were genetically related to sequences recovered in France.

### Time origins of E-5, E-9, E-18, and CV-A9 lineages

The lineages with pp>0.90 were numbered sequentially within the genogroups to analyze the evolutionary patterns of each EV type. E-5 genogroup d included most of the recent sequence data and arose in 2000 (TMRCA 95% HPD interval, 1995–2004) (**Fig 1B**). The sequences recovered in Tunisia clustered with sequences from France within the E-5/d3 genogroup, a lineage which split 10 years later (2010, 2008–2011). The TMRCA of genogroup E9/c was estimated in 1992 (1989–1995) (**Fig 2B**). Closely related E9 strains sampled in France and Tunisia belonged to the same genogroup d, which arose in 2000 (1997–2003). The E-18/c genogroup emerged in 1988 (1981–1993) and subsequently spread worldwide in three lineages dated between 1992 and 2000 (**Fig 3B**). The E-18/c3 genogroup included strains recovered in France and Tunisia, and was dated in 2000 (1998–2003). The TMRCA of the CV-A9 major lineage, genogroup i, was estimated in 1990 (1984–1994) (**Fig 4B**). The virus strains sampled in France and Tunisia clustered in genogroup i1 (1995, 1993–1997).

### Phylogenetic patterns of E-5, E-9, E-18, and CV-A9 inferred with 3CD sequence data

The individual lineages estimated with the 3CD gene phylogenetic tree clustered within three major clades that split from one another >200 years before 2013. The phylogenetic patterns of E-5, E-9, E-18, and CV-A9 genogroups in the 3CD tree exhibited major differences in comparison to those in the individual 1D/VP1 trees (**S1 Fig**).

Of the four EV types examined, E-18 had the most complex phylogenetic patterns because they displayed evidence of both ancient and recent recombination, and recent events resulted from both intertypic and intratypic recombination (**S1A Fig**). Ancient recombination was identified as the c2 genogroup partitioned into three distinct lineages within the 3CD clades 1 (n = 1 lineage) and 3 (n = 2). Similarly, two E-18/c3 lineages split in the two distant 3CD clades 2 and 3 (the latter being the E-18 Tunisian lineage), a pattern which indicated ancient recombination events. Comparison of phylogenies provided evidence of recent intertypic recombination between E-18/c3 strains co-circulating with strains of two E-6 and one CV-A9 lineages. Partitioning of a single E-18/c2 sequence within the E-18/c3 lineage showed recent intratypic recombination.

The 1D/VP1 and 3CD phylogenetic patterns for the other EV types indicated ancient recombination events. The E-5 genogroups c and d3 segregated into the same 3CD clade 1 but in distantly related lineages that had no direct temporal link (**S1B Fig**). The E9 genogroup b segregated into three distinct 3CD lineages, one in clade 1 and two in clade 3 (**S1C Fig**). Five phylogenetic patterns indicative of ancient recombination events were also identified in CV-A9 (**S1D Fig**): the h1 genogroup partitioned into two distinct lineages within the 3CD clade 1 and the i1 genogroup segregated into three lineages within clades 2 (n = 1) and 3 (n = 2).

Two phylogenetic patterns of recent recombination events were identified in E-9 and CV-A9 trees. Partitioning of an E9/c sequence within the E9/d lineage indicated an intratypic recombination event dated between 2005 (2000–2008) and 2009 (2007–2011). The other pattern showed intertypic recombination between CV-A9/i1 and E-18/c3 sequences.

### Virus migration patterns inferred with complete (near-complete) sequence samples

The geographic origins were inferred at all nodes of the 1D/VP1 tree to investigate virus migration between countries, and the changes in geographic locations between nodes and branches are shown by different colors (**Figs 1B, 2B, 3B and 4B**). Virus transportation was also estimated with the 3CD data (**S2 Fig**). Only the most probable changes estimated between closely related nodes in the two analyses were recorded (**Table 1**). Two consistent virus transportation events (noted T1 and T2) were determined between France and Tunisia from the E-5 genealogy (**Fig 1B**); the countries are indicated by alphabetical order. These transportation events were confirmed in the 3CD tree with probabilities of 1 and 0.99, respectively **(Table 1)**. Three virus transportation events (France/Tunisia, n = 2; Australia/France, n = 1) were estimated with the E-9 1D/VP1 tree (T3 to T5, **Fig 2B**). The 3CD tree confirmed a transportation event between France and Tunisia with a probability of 1. Virus spread between Australia and France was not confirmed as the 3CD sequence of the Australian virus was not available.

**Table 1:** Transportation events inferred from genealogies of four enterovirus types.

Virus transporation event	Enterovirus type	1D/VP1 gene			Partial 3CD gene		
		Location 1 (p; pp) ^a^	Location 2 (p; pp)^a^	Difference between TMRCA values (years) ^b^	Location 1 (p; pp) ^a^	Location 2 (p; pp) ^a^	Difference between TMRCA values (years)
T1	E-5	FRA (0.79; 1)	TUN (0.86; 1)	0.99	FRA (1; 1)	TUN (1; 1)	Overlap
T2	E-5	FRA (0.96; 1)	TUN (0.72; 1)	0.56	FRA (0.99; 1)	TUN (1; 1)	Overlap
T3	E9	FRA (0.67; 1)	TUN (0.54; 1)	0.2	FRA (1; 1)	TUN (0.99; 0.97)	Overlap
T4	E9	FRA (1; 1)	TUN (0.51; 0.94)	0.81			
T5	E9	AUS (0.96; 1)	FRA (0.96; 0.98)	0.77	ND	ND	ND
T6	E-18	AUS (1; 1)	FRA (0.81; 0.99)	0.68	ND	ND	ND
T7	E-18	AUS (0.92; 1)	FRA (0.82; 1)	0.75	ND	ND	ND
T8	CV-A9	CAN (1; 1)	CHN (0.92; 1)	0.42	ND	ND	ND
T9	CV-A9	FRA (0.97; 1)	TUN (0.95; 1)	0.22 Overlap	FRA (1; 1)	TUN (0.99; 1)	Overlap
T10	E6	ND	ND	ND	FRA (1; 1)	TUN (1; 1)	1.57

^a^ p, probability of the geographic location estimated at the relevant node; pp, posterior probability estimated for the same node.

^b^ The overlap of the 95% highest posterior probability (HPD) intervals estimated for the TMRCA of the two nodes is indicated.

Abbreviations: ND, not determined; TMRCA, time of the most recent common ancestor.

**Fig 5:**
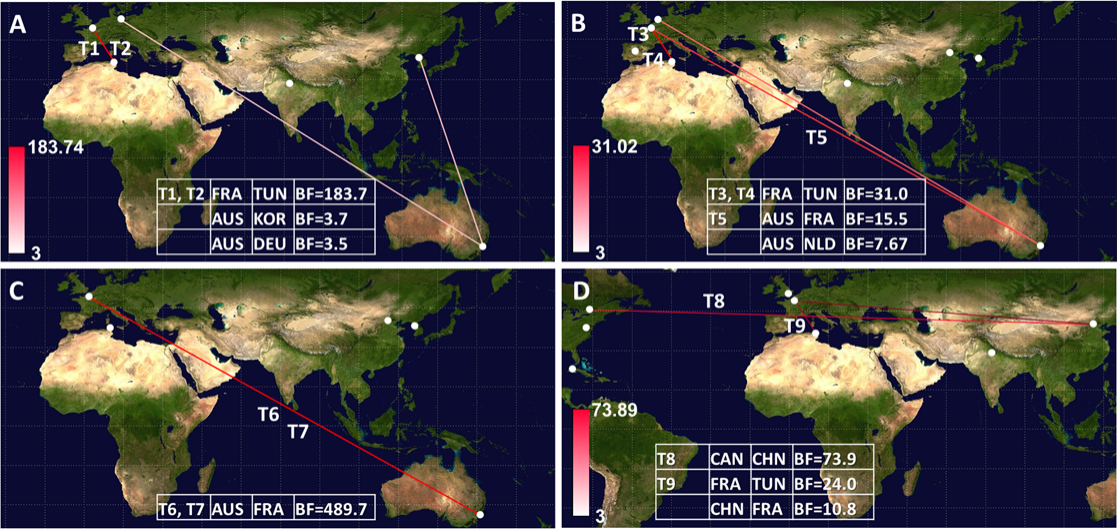
Spatial diffusion events supported with BF values > 3 for echovirus 5 (A), echovirus 9 (B), echovirus 18 (C), and coxsackievirus A9 (D). The phylogenetic analyses were performed with the samples of complete or near-complete 1D/VP1 gene sequences. The countries inferred to explain the probable diffusion events and the BF values are indicated within the inset tables. The countries are listed alphabetically; the order of country names does not indicate the direction of the spatial migration. The sampling countries are indicated by full white circles. The probable spatial migration events T1 to T9 are shown. The lines connecting countries are colored according to the intensity scale (shown in panel A), which indicates increasing BF values (range from 3 to 500).

Two virus transportations involving Australia and France were estimated with the E-18 phylogenetic data (T6 and T7 in **Fig 3B**). Although the E-18 sequences sampled in Tunisia in 2013 displayed close phylogenetic relatives among the viruses sampled in France, the 1D/VP1 phylogenetic data provided no evidence of recent virus transportation between the two countries (difference between the 95% HPD intervals of TMRCA values for the inferred nodes = 5.7 years). Two phylogenetic patterns of recent virus transportation were estimated in the 1D/VP1 CV-A9 genealogy: T8, Canada/China and T9, France/Tunisia (**Fig 4B**). The second event was confirmed in the analysis of 3CD sequences. Finally, the T10 transportation event between France and Tunisia was estimated with a probability of 1 with the 3CD phylogenetic tree. The virus involved was an E-6 strain, as determined by the 1D/VP1 sequence (**data not shown**). The spatial diffusion rates (BSSVS estimates) calculated between sampling locations were analyzed with a BF test to investigate whether the virus transportation events assessed empirically from the MCC trees were supported statistically. The transportation patterns supported by BF threshold > 3 are given in **Fig 5**. The nine virus transportation events assessed with the 1D/VP1 datasets were supported by BF values > 10, a value which was used as a threshold in the analyses described below. The E-5 and E-9 phylogenetic data provided indication of three additional transportation events supported by lower BF values.

### Virus migration patterns inferred with large partial 1D/VP1 sequence samples

**Fig 6:**
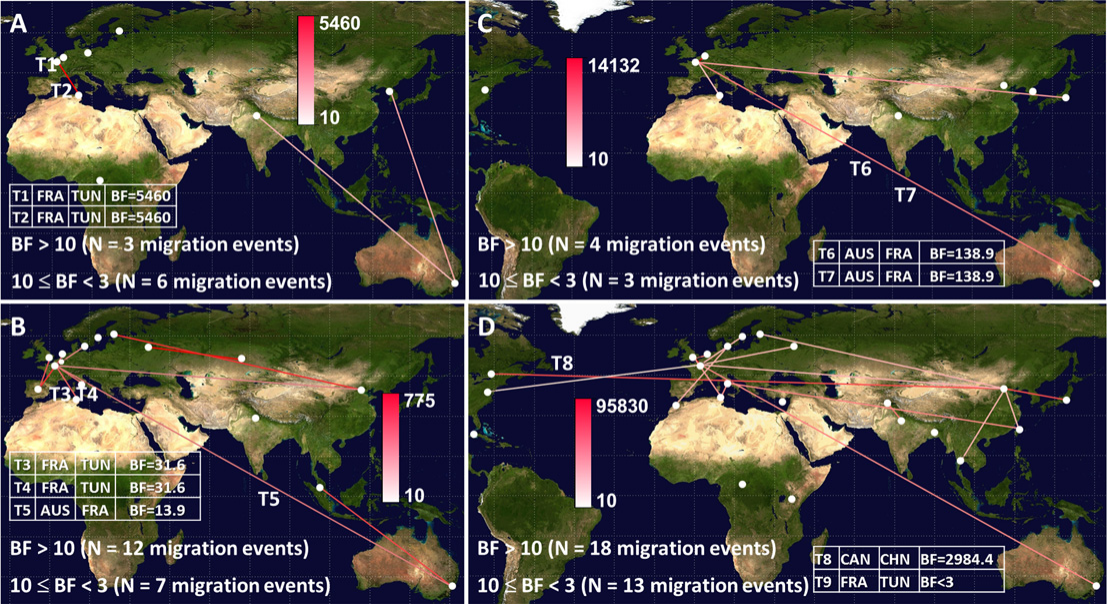
Spatial diffusion patterns assessed for echovirus 5 (A), echovirus 9 (B), echovirus 18 (C), and coxsackievirus A9 (D). The diffusion patterns were inferred through phylogenetic analyses of sequence samples derived from the 5’ part of the 1D/VP1 gene. The sampling countries are indicated with full white circles. The lines connecting countries were colored according to the intensity scales indicating increasing BF values shown within each panels. The complete lists of spatial migration events with BF values > 10 are given in S4 Table.

The confidence level in the spatial diffusion events estimated between France and Tunisia was further investigated with phylogenetic data derived from sequence samples (5’ part of the 1D/VP1 gene) larger than those used in the investigation reported above. The Bayes factor values calculated for these analyses are shown in **S4 Table**. The phylogenetic data inferred with the large E-5 sequence sample supported three spatial diffusion events (BF values > 10), of which one was a transportation event between France and Tunisia (**Fig 6A**). The large E-9 partial sequence dataset provided evidence of 12 spatial migrations supported by BF values > 10 (**Fig 6B**); the analysis confirmed the transportation events between France and Tunisia. Seven virus migration events were also supported by BF values ranging from 3 to 10 (**data not shown**). Four spatial migration events were assessed by analysis of the large E-18 partial sequence sample (**Fig 6C**). A virus migration pattern inferred between Australia and France (BF = 138.9) confirmed the T6 and T7 transportation events. A migration between France and Tunisia, not detected by analysis of the small sequence sample, was supported by a BF value = 11.5. The CV-A9 diffusion pattern comprised 18 spatial migrations supported by BF values > 10 and confirmed two out of three spatial diffusion events (including T8) inferred with the near-complete sequence sample (**Fig 6D**). The T9 virus migration between France and Tunisia was not supported.

Sequence samples derived from the 3’ part of the E-18 and CV-A9 1D/VP1 genes were analyzed to investigate further the discrepancies in the above analyses: additional virus migration between France and Tunisia and low support of the T9 virus transportation (**S3 Fig**). A control analysis with an E-9 sequence sample showed that the T3, T4, and T5 events were supported by BF > 10 (**S3A Fig**), as they were in the analyses of the other two sequence samples (**Fig 5A**, **Fig 6A**). The sample of nt sequences derived from the 3’ part of the E-5 gene was too small to be used as a control. The analysis of the E-18 sample (**S3B Fig**) supported virus migration between Australia and France (BF = 12), thereby strengthening the confidence in the T6 and T7 events. The virus migration between France and Tunisia was supported by a BF = 7. The CV-A9 phylogenetic data (**S3C Fig**) gave support to ten spatial migrations, two of which confirmed the T8 and T9 virus transportation events (Canada/China, BF = 660; France/Tunisia, BF = 270).

## Discussion

In this study, we used a phylodynamic approach to map the geographic movements of virus strains among four EV types. The phylogenetic data inferred from 1D/VP1 sequence samples showed a large array of virus migrations across countries, some of which assessed recent virus transportation events between France and Tunisia. The E-5, E-9, and CV-A9 phylogenetic patterns provided evidence of virus migrations between the two countries, while the E-18 phylogenetic data were less consistent. The migration patterns reconstructed for CV-A9 provided evidence for virus migration between different European countries and Tunisia in a recent past. The phylogeny reconstructed for a same lineage of closely related CV-A9 strains supported virus migrations between Tunisia and three other countries (France, Italy, and the United Kingdom), a pattern which suggests frequent and complex virus movements between European countries and Tunisia. Factors such as close economic relationships and tourism flows, which promote frequent displacement of individuals, may be involved. For instance nearly 98,000 French nationals were living in North Africa in December 2012 [**49**] and tourists from France represented 1/6 of those visiting Tunisia before 2011 [**50**]. This hypothesis should be examined with larger sequence samples.

**Fig 7:**
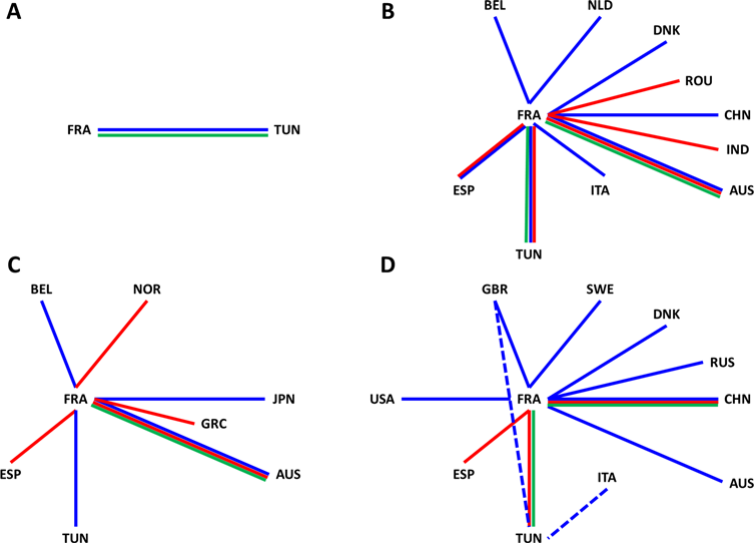
Schematic representation of the connections between France, Tunisia, and other countries assessed with BF > 10 from the phylogenetic patterns of echovirus 5(A), echovirus 9(B), echovirus 18 (C), and coxsackievirus A9 (D). The figure summarizes the data shown in Figs 5 and 6 and S3 Fig. The connections were estimated from the complete or near-complete 1D/VP1 gene sequences (green lines) and the partial sequences derived from the 5’ (blue lines) and 3’ (red lines) part of the 1D/VP1 gene. The solid lines indicate connections between France and other countries and dashed lines those between Tunisia and other countries.

The phylogeographic patterns were influenced by missing data, a common bias present in sequence samples [**51**]. The spatial diffusion patterns inferred were dependent on the sequence datasets devised for a given EV type (**Fig 7**). The conservative method used for assessing the most likely virus transportation events that occurred between countries over short periods was robust to this sample bias. All the virus transportation events assessed with the empirical method exhibited BFs > 10, a value which was used as a threshold to select the most probable virus migrations. Phylogenetic data inferred from non-structural genes provided further support of virus migrations: the analysis of 3CD gene suggested a migration event between France and Tunisia involving strains of the EV type E-6. The overall analyses of the four EV types showed that France was connected to a range of 1 to 10 countries including Tunisia: the larger the size of the sequence sample analyzed, the higher the number of countries. A survey of 156 French patients with EV meningitis reported between 2008 and 2013 showed that only 2.6% of cases were caused by E-5 and E-9 [**34**]. In the same survey, CV-A9 and E-18 infections were reported more frequently (5.8% and 7.1% of meningitis cases, respectively). This epidemiological pattern of infrequent infections is in agreement with the phylogenetic data, which suggest occasional transfers of virus strains between France and other countries.

The present study provides phylogenetic evidence of earlier hypotheses suggesting that different lineages of a given EV type may have different transmission features and geographic distributions. The EV type E-30 genogroup VI (defined by Ke et al [**52**], also designated lineages d to h in [**53**]) has been reported in multiple worldwide outbreaks of acute meningitis over the last 15 years, in contrast to the re-emerging genogroup III (lineage a), which exhibited ‘endemic features’ in Russia [**54**]. Similar observations were derived from molecular epidemiology studies of two other infrequent EV types, E-3 and CV-B1. In E-3, all genogroups were isolated worldwide but genogroup C was more frequently isolated in acute flaccid paralysis in India [**55**]. In CV-B1, genogroup IV has a wide geographic distribution compared to genogroup III, which has been more frequently reported in India [**56,57**]. Similarly, our phylogenetic data for E-9 showed that a genogroup c virus was spread between France and Australia while a few years later genogroup d strains were involved in virus migration between France and Tunisia. The varying feature of CV-A9 showed that distinct but closely related strains of the same genogroup i1 were involved in virus migrations between different countries, at about the same period. The epidemic and endemic patterns seen in different EV types might be related to these differences in virus transportation but other features could also be involved (see below).

The overall data reported in this study have additional interest beyond estimations of a number of virus migration events between different countries: they are evidence that EV phylogenies had no strong spatial structure even over short periods of time. The global persistence of EV lineages and the local seasonal recurrence of infections are underlined by worldwide transportation of virus strains, as seen for instance in human influenza A [**58**]. The four EV types were widely distributed over the world despite being infrequently reported in human diseases [**28,59**]. The main genogroup of each EV type (i.e. the genogroup including the majority of the most recently sampled strains) was reported in a range of three to five continents. In this respect, CV-A9 was similar to the other three types although it was considered as an endemic EV in the USA [**28**]. In addition, CV-A9 and E-9 shared a common feature as they were phylogenetically structured into a greater number of genogroups (n = 10 and 5, respectively) in comparison to the other two types (n = 3 and 4). An earlier study reported 12 CV-A9 genogroups by analysis of the partial VP1-2A genomic region [**37**]. More specifically, CV-A9 and E-9 differed from the other two types by the number of epidemiologically active genogroups. Their phylogenies showed that the infections reported over the last 20 years resulted from the worldwide co-circulation of lineages among five and four genogroups respectively. In contrast, the E-5 and E-18 infections mainly arose from the co-circulation of lineages of one predominant genogroup. Whether or not these variations in co-circulation are involved in endemic or epidemic features will need to be established for a greater number of EV types.

Geographic distance was not a barrier to virus transmission of EV strains as transportations of E-5, E-9, E-18, and CV-A9 were observed between distant countries. Virus migration is necessary but not sufficient for a virus lineage to initiate a successful transmission chain in the receiver population. The epidemiological consequences of virus transportation are also determined by herd immunity against the imported virus strain. Future research is needed on other infrequently reported (EV-D68) and epidemic (E-30 and EV-A71) EV types to determine which factor among virus transportation and local virus transmission is involved in the occurrence of outbreaks.

## Supporting Information

S1 Fig Incongruence between tree topologies inferred with the 1D/VP1 and 3CD gene sequences for enterovirus types E-18 (S1A), E-5 (S1B), E-9 (S1C), and CV-A9 (S1D). The phylogenetic clusters in the 1D/VP1 trees (left panels) were traced back to the 3CD tree (right panels) to investigate the involvement of recombination in the evolutionary pathway of each EV type. The 1D/VP1 trees are monophyletic (all the lineages within a single tree derived from a common ancestor assigned to the same type) while the lineages within the 3CD phylogeny are polyphyletic (they share several ancestors assigned to different types). The 3CD tree was inferred with 117 sequences from five EV types, those analyzed in the study plus E-6.
(PDF)

S2 Fig Phylogenetic tree inferred with 3CD sequence data of five enterovirus types (E-5, E-6, E-9, E-18, and CV-A9) showing virus transportation events. The phylogeny was estimated with 117 3CD gene sequences by a Bayesian Markov chain Monte Carlo analysis implementing a discrete phylogeographic model. The branches are colored according to the geographic location that had the highest probability; the color code is indicated in the Figure. The tree nodes are indicated with circles colored according to the most probable geographic location; circle size is proportional to probability. A node shown with an open dark circle (and letter T followed by a number) indicates the source of a transportation event of a virus strain between two different countries (see also Table 1). The scale at the bottom of the figure indicates calendar years. Abbreviations used: CAN, Canada; CUB, Cuba; FRA, France; KOR, Korea; NLD, Netherlands; TUN, Tunisia; USA, United States of America.
(TIF)

S3 Fig Spatial diffusion patterns assessed for echovirus 9 (A), echovirus 18 (B), and coxsackievirus A9 (C). The diffusion patterns were inferred through phylogenetic analyses of sequence samples derived from the 3’ part of the 1D/VP1 gene. The alignments were constructed by selecting the largest number of sequences that shared as many nucleotide positions as possible within the 3’ part of the 1D/VP1 gene. The general features of the sequence datasets used are indicated on each panel. The nucleotide positions common to all partial sequences are indicated (the numbering refers to the following reference sequences: CV-A9, D00627; E-5, AF083069; E-9, AF524866; and E-18, AF317694). The sampling countries are indicated with full white circles. The lines connecting countries were colored according to the intensity scales indicating increasing BF values. The virus transportation events assessed with the complete (or near-complete) sequence samples are indicated.
(TIF)

S1 Table Enterovirus samples used in the study. (PDF)

S2 Table Publicly available sequence data used in the study. (PDF)

S3 Table General features of the 1D/VP1 gene sequence datasets used in the study. (DOCX)

S4 Table Spatial diffusion assessed with the phylogenetic data inferred from the 5' partial 1D/VP1 sequences of four enterovirus types and comparison with the spatial diffusion assessed with other sequence datasets. (DOCX)
