# Supplementary material for: Enterovirus Migration Patterns between France and Tunisia
Source: PLoS One. 2015 Dec 28;10(12):e0145674. doi: 10.1371/journal.pone.0145674 (PMC4692522; doi:10.1371/journal.pone.0145674)

Supplementary Figure 1A

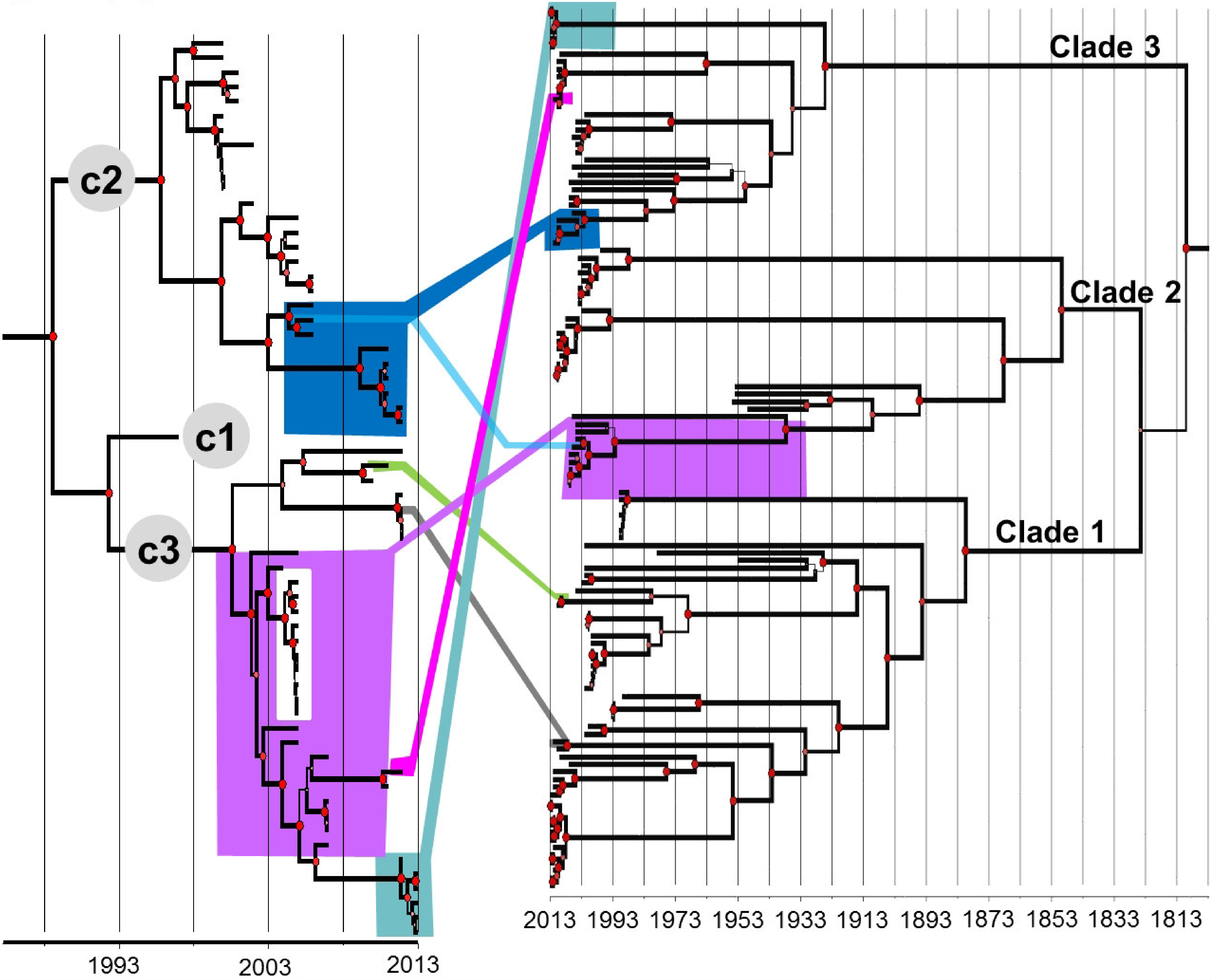

Supplementary Figure 1B

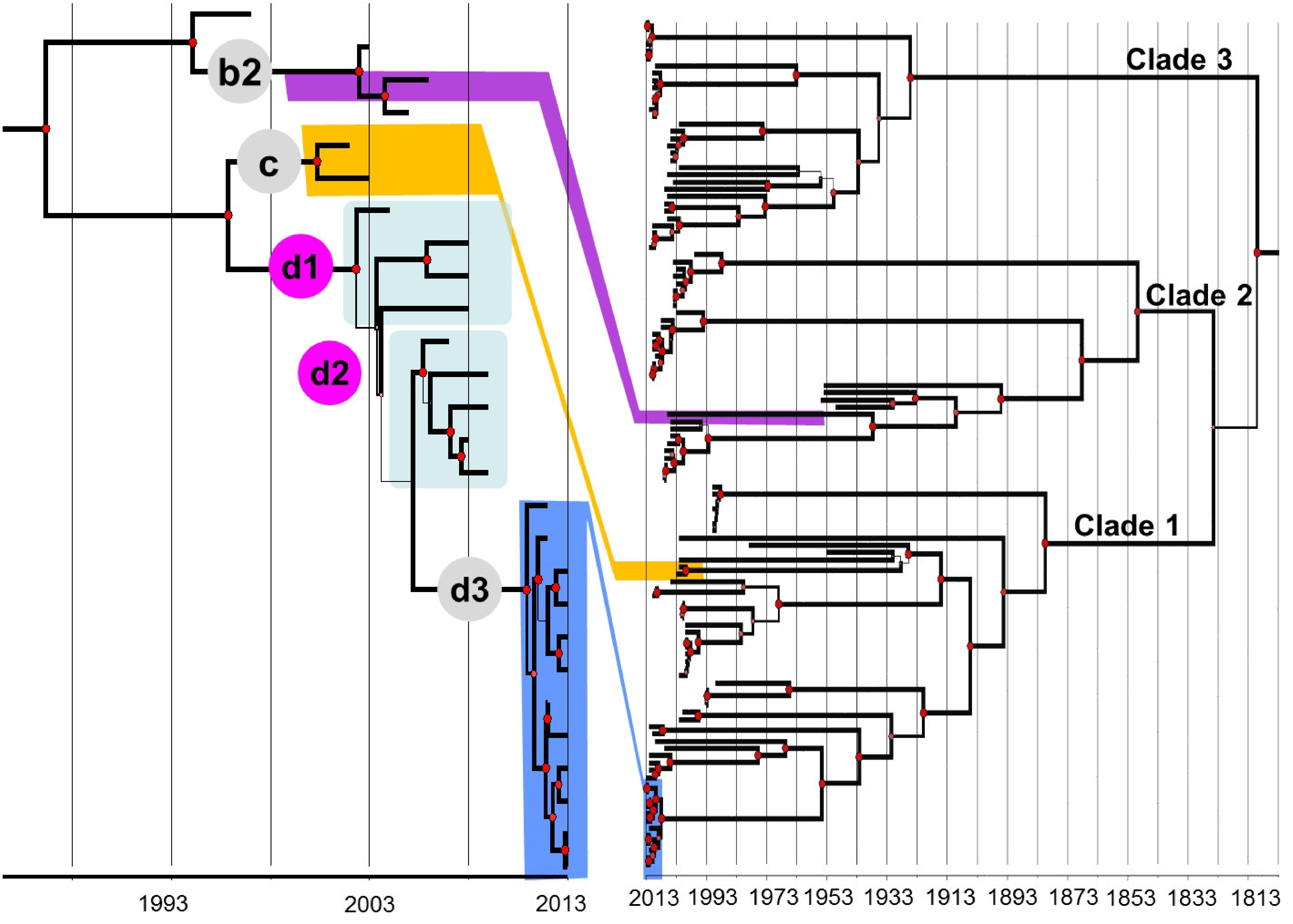

Supplementary Figure 1C

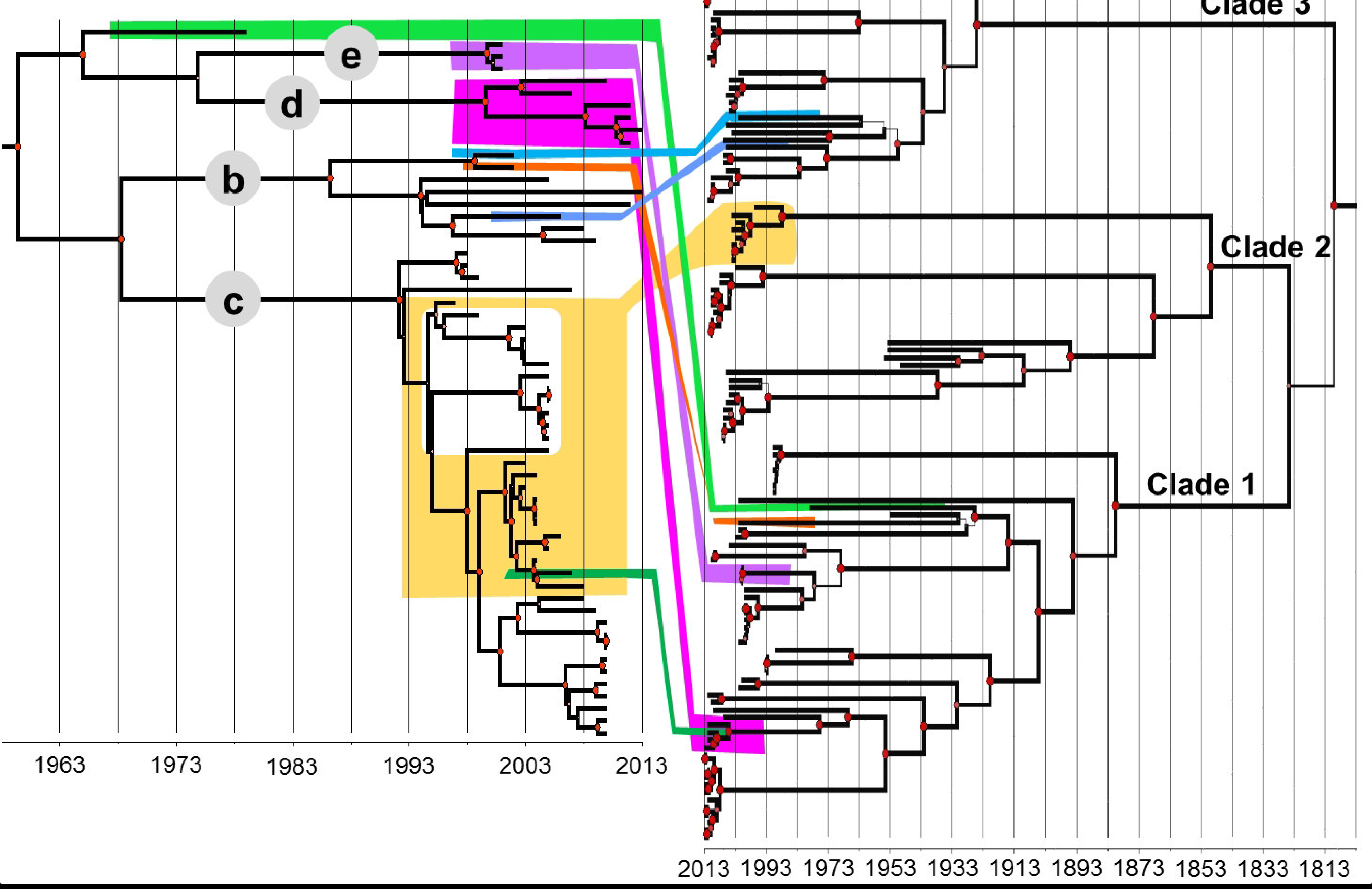

Supplementary Figure 1D

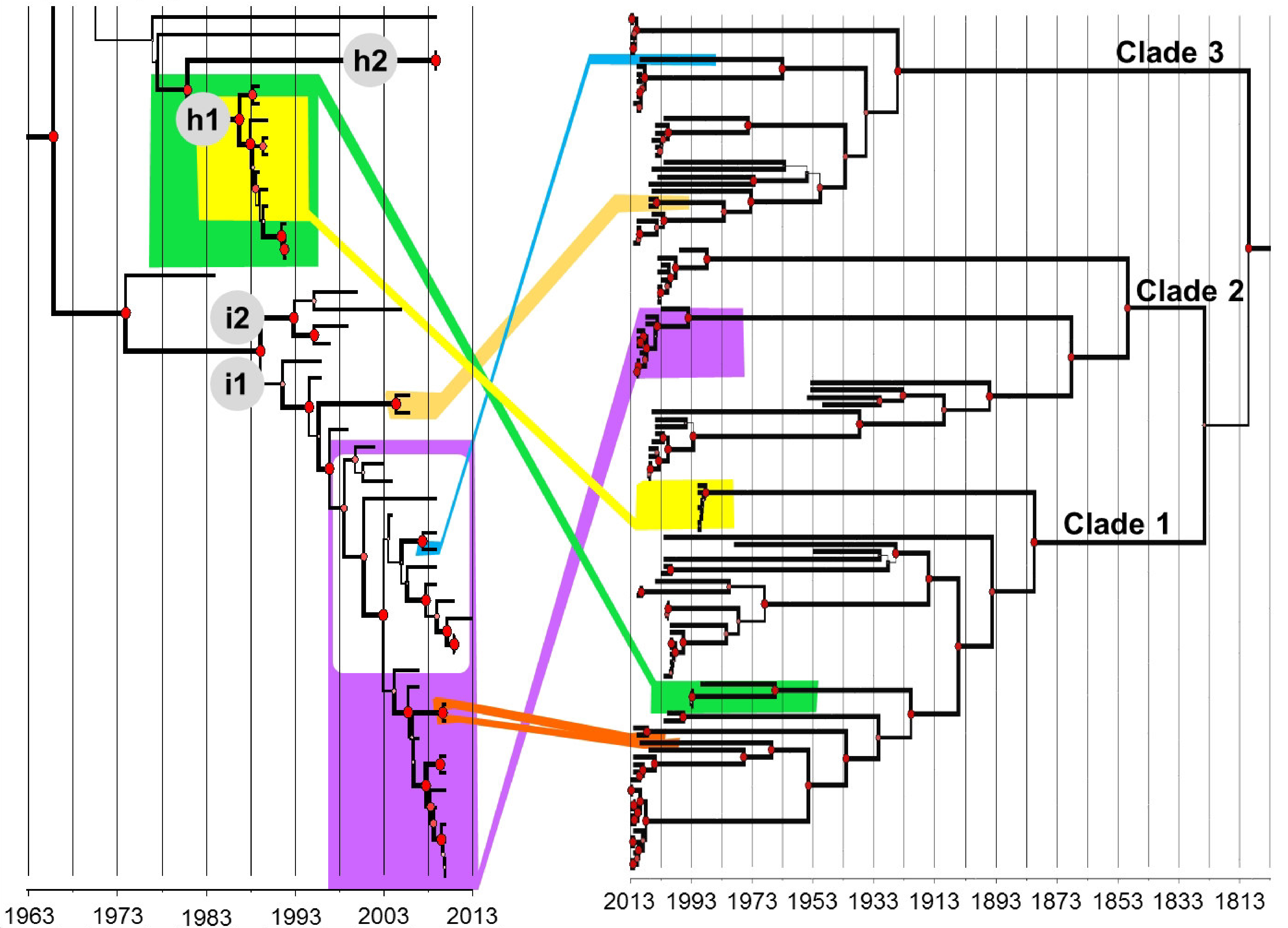

Supplement: S1 Fig — The phylogenetic clusters in the 1D/VP1 trees (left panels) were traced back to the 3CD tree (right panels) to investigate the involvement of recombination in the evolutionary pathway of each EV type. The 1D/VP1 trees are monophyletic (all the lineages within a single tree derived from a common ancestor assigned to the same type) while the lineages within the 3CD phylogeny are polyphyletic (they share several ancestors assigned to different types). The 3CD tree was inferred with 117 sequences from five EV types, those analyzed in the study plus E-6. (PDF) [file pone.0145674.s001.pdf]
