# Supplementary figures and images for: Enterovirus Migration Patterns between France and Tunisia
Source: PLoS One. 2015 Dec 28;10(12):e0145674. doi: 10.1371/journal.pone.0145674 (PMC4692522; doi:10.1371/journal.pone.0145674)

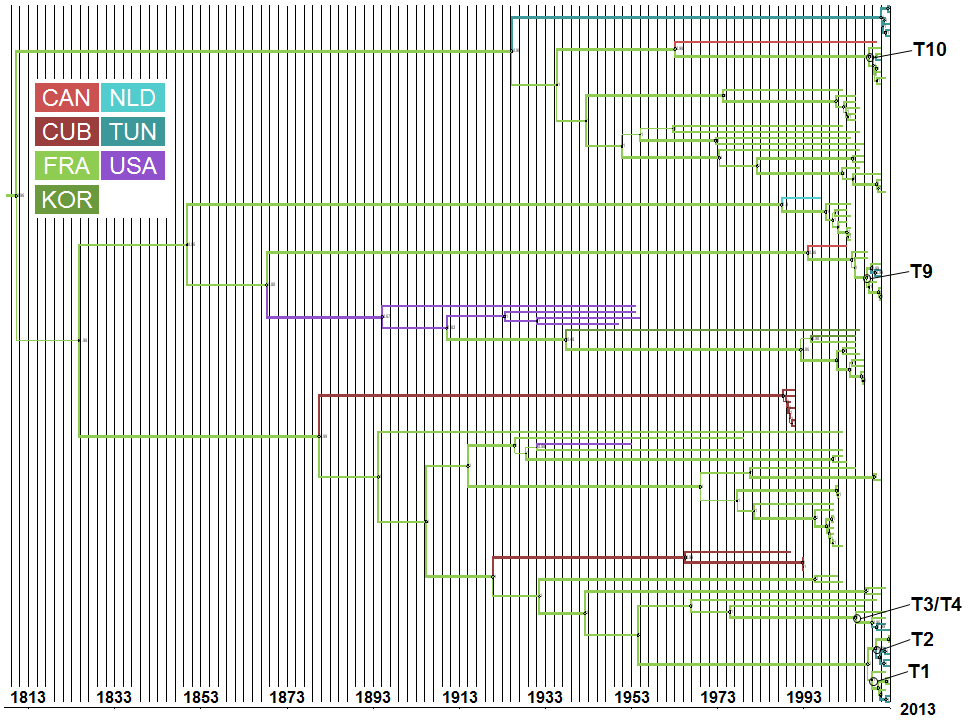

Supplement: S2 Fig — The phylogeny was estimated with 117 3CD gene sequences by a Bayesian Markov chain Monte Carlo analysis implementing a discrete phylogeographic model. The branches are colored according to the geographic location that had the highest probability; the color code is indicated in the Figure. The tree nodes are indicated with circles colored according to the most probable geographic location; circle size is proportional to probability. A node shown with an open dark circle (and letter T followed by a number) indicates the source of a transportation event of a virus strain between two different countries (see also Table 1). The scale at the bottom of the figure indicates calendar years. Abbreviations used: CAN, Canada; CUB, Cuba; FRA, France; KOR, Korea; NLD, Netherlands; TUN, Tunisia; USA, United States of America. (TIF) [file pone.0145674.s002.tif]

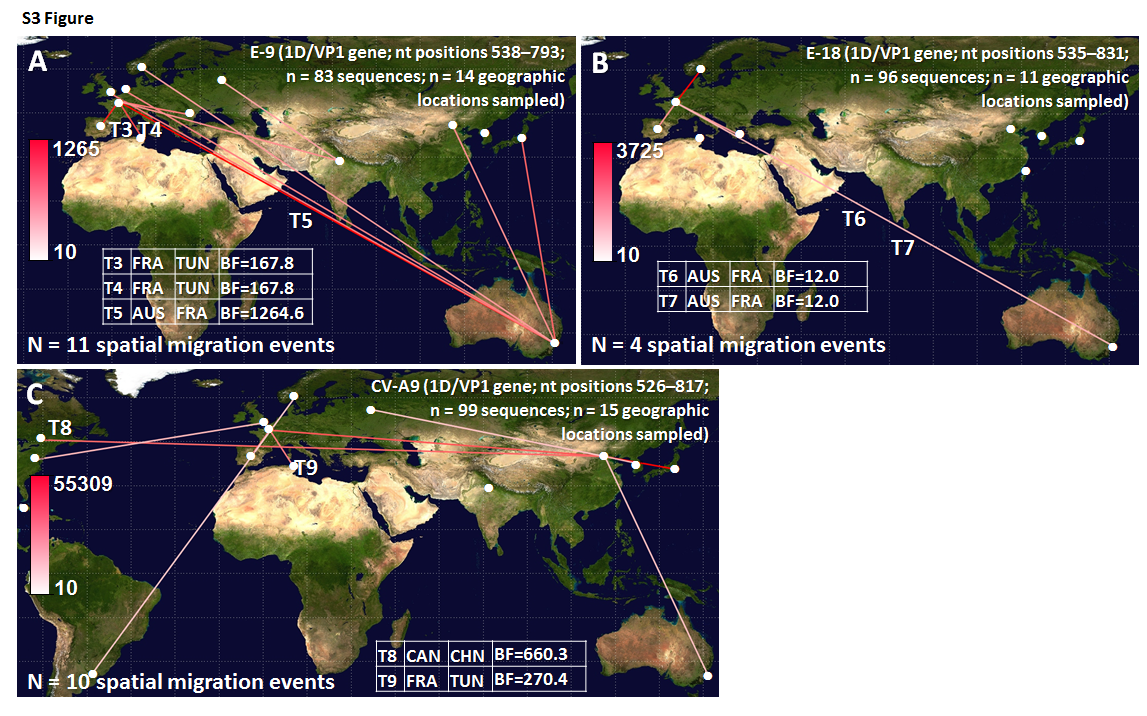

Supplement: S3 Fig — The diffusion patterns were inferred through phylogenetic analyses of sequence samples derived from the 3’ part of the 1D/VP1 gene. The alignments were constructed by selecting the largest number of sequences that shared as many nucleotide positions as possible within the 3’ part of the 1D/VP1 gene. The general features of the sequence datasets used are indicated on each panel. The nucleotide positions common to all partial sequences are indicated (the numbering refers to the following reference sequences: CV-A9, D00627; E-5, AF083069; E-9, AF524866; and E-18, AF317694). The sampling countries are indicated with full white circles. The lines connecting countries were colored according to the intensity scales indicating increasing BF values. The virus transportation events assessed with the complete (or near-complete) sequence samples are indicated. (TIF) [file pone.0145674.s003.tif]
