# Supplementary material for: Enterovirus Migration Patterns between France and Tunisia
Source: PLoS One. 2015 Dec 28;10(12):e0145674. doi: 10.1371/journal.pone.0145674 (PMC4692522; doi:10.1371/journal.pone.0145674)
Supplement: S1 Table — (PDF) [file pone.0145674.s004.pdf]

S1 Table. Enterovirus sample used in the study.

Enterovirus type	Isolate designation	Country of isolation	Year of isolation	1D/VP1 accession number	3CD accession number
E-18	CF1400191	France	2005	AM236918	LN876214
E-18	TR115015	France	2005	AM236970	LN876215
E-18	TR129002	France	2005	AM236972	LN876216
E-18	TR157051	France	2005	AM236978	LN876217
E-18	TR178027	France	2005	AM236984	LN876218
E-18	J3	Tunisia	2013	LN876153	LN876219
E-18	J6	Tunisia	2013	LN876154	LN876220
E-18	S4	Tunisia	2013	LN876155	LN876221
E-18	P13	Tunisia	2013	LN876156	LN876222
E-18	S9	Tunisia	2013	LN876156	LN876223
E-18	M3	Tunisia	2012	LN713457	LN876224
E-18	CF138049	France	2011	LN876158	LN876225
E-18	CF018113	France	2012	LN876159	LN876226
E-18	CF222019	France	2012	LN876160	LN876227
E-18	CF058098	France	2012	LN876161	LN876228
E-18	CF705	France	2000	LN876162	LN876229
E-18	CF1312	France	2000	LN876163	LN876230
E-18	CF1440	France	2000	LN876164	LN876231
E-18	CF1801	France	2000	LN876165	LN876232
E-18	CF1402	France	2002	LN876166	LN876233
E-18	CF159050	France	2007	LN876167	LN876234
E-18	CF171003	France	2007	LN876168	LN876235
E-18	CF171129	France	2007	LN876169	LN876236
E-18	CF188018	France	2007	LN876170	LN876237
E-18	CF190038	France	2007	LN876171	LN876238
E-18	TR185035	France	2005	LN876172	LN876239
E-18	CF303024	France	2006	AM711105	LN876240
E-18	CF217037	France	2011	HG793719	LN876241
E-18	CF332118	France	2011	HG793722	LN876242
E-18	CF339116	France	2011	HG793723	LN876243
CV-A9	CF185079	France	2007	LN876177	LN876244
CV-A9	F26	Tunisia	2011	LN713452	LN876245
CV-A9	F29	Tunisia	2011	LN713453	LN876246
CV-A9	CF152050	France	2010	LN876173	ND
CV-A9	CF035013	France	2011	LN876174	LN876247
CV-A9	CF193068	France	2011	LN876175	LN876248
CV-A9	CF215004	France	2011	LN876176	ND
CV-A9	CF192070	France	2011	HG793661	LN876249
CV-A9	CF197008	France	2011	HG793663	LN876250
CV-A9	CF207004	France	2011	HG793664	LN876251
CV-A9	CF179026	France	2010	HG793658	LN876252
CV-A9	CF186011	France	2008	HG793656	LN876253
CV-A9	CF239034	France	2008	HG793657	LN876254
CV-A9	TR081056	France	2005	AM236967	LN876255
E-5	M7	Tunisia	2012	LN713451	LN876178
E-5	L355	Tunisia	2013	LN713449	LN876179
E-5	P3	Tunisia	2013	LN876128	LN876180
E-5	P2	Tunisia	2013	LN876129	LN876181
E-5	P11	Tunisia	2013	LN876130	LN876182
E-5	S2	Tunisia	2013	LN876131	LN876183
E-5	CF206019	France	2012	HG793680	LN876184
E-5	CF203016	France	2012	HG793679	LN876185
E-5	CF204030	France	2013	LN876132	LN876186
E-5	CF259002	France	2013	LN876133	LN876187
E-5	CF192042	France	2013	LN876134	LN876188
E-5	CF224001	France	2013	LN876135	LN876189
E-5	CF676	France	2002	LN876136	LN876190
E-5	CF262037	France	2003	LN876137	LN876191
E-9	L363	Tunisia	2012	LN713460	LN876199
E-9	P6	Tunisia	2013	LN876138	LN876200
E-9	CF215010	France	2010	LN876139	LN876201
E-9	CF180023	France	2012	LN876140	LN876202
E-9	CF220033	France	2012	LN876141	LN876203
E-9	CF362040	France	2013	LN876142	ND
E-9	CF1140	France	1979	LN876143	LN876204
E-9	CF1327	France	2001	LN876144	LN876205
E-9	CF1434	France	2001	LN876145	LN876206
E-9	CF788	France	2002	LN876146	LN876207
E-9	CF269061	France	2003	LN876147	LN876208
E-9	CF317080	France	2003	LN876148	LN876209
E-9	CF164002	France	2004	LN876149	LN876210
E-9	CF169089	France	2004	LN876150	LN876211
E-9	CF190030	France	2004	LN876151	LN876212
E-9	CF356122	France	2004	LN876152	LN876213

The nucleotide sequences determined in the present study are shown by accession numbers indicated in red.
The nucleotide sequences determined in previous studies are shown by accession numbers indicated in black.
ND, not determined.
