# Supplementary material for: Enterovirus Migration Patterns between France and Tunisia
Source: PLoS One. 2015 Dec 28;10(12):e0145674. doi: 10.1371/journal.pone.0145674 (PMC4692522; doi:10.1371/journal.pone.0145674)
Supplement: S2 Table — (PDF) [file pone.0145674.s005.pdf]

S2a Table. Publicly available sequence data for echovirus 5 used in the study.

N	Isolate name	Country of isolation	Year of isolation	1D/VP1 accession number	3CD accession number
1	Noyce	united state	1954	AF083069	AF083069
2	N_173	India	2007	JN203687	ND
3	N_468	India	2008	JN203688	ND
4	N_487	India	2008	JN203689	ND
5	N_713	India	2008	JN203690	ND
6	N_802A	India	2009	JN203691	ND
7	04_131_2665	Australia	2004	FJ868338	ND
8	05_242_3696	Australia	2005	FJ868339	ND
9	97_070_0344	Australia	1997	GU142878	ND
10	KCD75	India	2009	JX513450	ND
11	KCD49A	India	2009	JX513451	ND
12	MR34	India	2008	JX513452	ND
13	Kor06_ECV5_253cn	Korea	2006	HM775882	HM775882

S2b Table. Publicly available sequence data for echovirus 9 used in the study.

N	Isolate name	Country of isolation	Year of isolation	1D/VP1 accession number	3CD accession number
1	Barty	United States of Americ	1953	AF524866	AF524866
2	DM	NLD	1997	AF524867	AF524867
3	Hangzhou_332_10	China	2010	KC182535	ND
4	Hangzhou_331_10	China	2010	KC182534	ND
5	98_330_1317	Australia	1998	GU142894	ND
6	98_357_2018	Australia	1998	GU142893	ND
7	99_046_2082	Australia	1999	GU142892	ND
8	99_082_1973	Australia	1999	GU142891	ND
9	07_318_0473	Australia	2007	GU142890	ND
10	03_177_1464	Australia	2003	GU142889	ND
11	07_262_0901	Australia	2007	GU232810	ND
12	06_065_4398	Australia	2006	FJ868342	ND
13	05_330_2064	Australia	2005	FJ868341	ND
14	04_349_2761	Australia	2005	FJ868340	ND
15	03_199_3019	Australia	2003	FJ868299	ND
16	E9_03_006_2652	Australia	2003	FJ868298_	ND
17	N_933	India	2009	JN203737	ND
18	N_460	India	2008	JN203736	ND
19	JB141090053	China	2009	KC867097	ND
20	JB14080073	China	2008	KC867096	ND
21	kor05_ECV9_245cn	Korea	2005	EU604665	ND
22	060_LS_CHN_AM_08	China	2010	KF246762	ND
23	051_LS_CHN_AM_08	China	2010	KF246761	ND
24	010_LS_CHN_AM_08	China	2010	KF246760	ND
25	074_LY_CHN_AM_10	China	2010	KF150162	ND
26	065_LY_CHN_AM_10	China	2010	KF150161	ND
27	063_LY_CHN_AM_10	China	2010	KF150160	ND
28	Kor05_ECV9_360cn	Korea	2005	EU590813	ND
29	Kor05_ECV9_352cn	Korea	2005	EU590812	ND
30	Kor05_ECV9_320cn	Korea	2005	EU590811	ND
31	Kor05_ECV9_296cn	Korea	2005	EU590810	ND
32	Kor05_ECV9_288cn	Korea	2005	EU590809	ND
33	MSH_KM812	China	2010	JN596587	ND
34	M10MF37	China	2010	JN655888	ND
35	ESP08_54628	Spain	2008	FR798004	ND

S2c Table. Publicly available sequence data for echovirus 18 used in the study.

N	Isolate name	Country of isolation	Year of isolation	1D/VP1 accession number	3CD accession number
1	Metcalf	United state	1955	AF317694	AF317694
2	05_220_3740	Australia	2005	GU142897	nd
3	00_220_3243	Australia	2001	GU142898	nd
4	01_090_1186	Australia	2001	GU142899	nd
5	01_092_3110	Australia	2001	GU142900	nd
6	01_267_3334	Australia	2001	GU142901	nd
7	97_113_0269	Australia	1997	GU142902	nd
8	CF506	France	2000	HF948104	HF948104
9	CF703	France	2000	HF948105	HF948105
10	CF1442	France	2002	HF948106	HF948106
11	CF272084	France	2006	HF948107	HF948107
12	CF287012	France	2006	HF948108	HF948108
13	05430_SD	China	2005	GQ329813	nd
14	247016	France	2006	AM711068	nd
15	303018	France	2006	AM711073	nd
16	kor05_ECV18_054cn	Korea	2005	EU604664	HM777023
17	04_090_3608	Australia	2004	FJ868347	nd
18	04_338_1946	Australia	2005	FJ868348	nd

19	04_356_3127	Australia	2005	FJ868349	nd
20	05_073_4094	Australia	2005	FJ868350	nd
21	05_206_3218	Australia	2005	FJ868351	nd
22	05_206_3225	Australia	2005	FJ868352	nd
23	05_207_4410	Australia	2005	FJ868353	nd
24	05_255_3084	Australia	2005	FJ868354	nd
25	05_165_3625	Australia	2005	FJ868306	nd
26	05_318_4308	Australia	2005	FJ868307	nd

S2d Table. Publicly available sequence data for coxsackievirus A9 used in the study.

N	Isolate name	Country of isolation	Year of isolation	1D/VP1 accession number	3CD accession number
1	Griggs_Bozek	United state	1950	D00627	D00627
2	FJ00_127	China	2000	AY573578	ND
3	FJ98_90	China	1998	AY573577	ND
4	CVA9_Alberta_2010	Canada	2010	JQ837914	JQ837914
5	Cuba689of93	Cuba	1993	AY466032	AY466032
6	Cuba47of93	Cuba	1993	AY466031	AY466031
7	Cuba35of93	Cuba	1993	AY466030	AY466030
8	Cuba267of90	Cuba	1990	AY466029	AY466029
9	Cuba163of91	Cuba	1991	AY466028	AY466028
10	Cuba135of91	Cuba	1991	AY466027	AY466027
11	Cuba100of91	Cuba	1991	AY466026	AY466026
12	Cuba45of91	Cuba	1991	AY466025	AY466025
13	Cuba450of90	Cuba	1990	AY466023	AY466023
14	Cuba96of91	Cuba	1991	AY466022	AY466022
15	Cuba270of90	Cuba	1990	AY466024	AY466024
16	CVA9_Alberta_2003	Canada	2003	JQ837913	JQ837913
17	CO62_GBR62	Great Britain	1962	JN996502	ND
18	CO79_GBR79	Great Britain	1979	JN996501	ND
19	CO85_GBR85	Great Britain	1985	JN996500	ND
20	CO87_GBR87	Great Britain	1987	JN996499	ND
21	04318_SD_CHN_2004	China	2004	GQ329731	ND
22	01332_SD_CHN_2001	China	2001	GQ329730	ND
23	00365_SD_CHN_2000	China	2000	GQ329729	ND
24	97186_SD_CHN_1997	China	1997	GQ329728	ND
25	97089_SD_CHN_1997	China	1997	GQ329727	ND
26	024_LS_CHN_AM_08	China	2010	KF246747	ND
27	071_LY_CHN_AM_10	China	2010	KF150146	ND
28	064_LY_CHN_AM_10	China	2010	KF150145	ND
29	CVA9_YZ047_SD_CHN_2005	China	2005	GQ246517	ND
30	A450D	India	2011	JX513563	ND
31	253_JN_CHN_AM_10	China	2010	KF246748	ND
32	99_358_0740	Australia	1999	GU142874	ND
33	06_109_3344	Australia	2006	FJ868282	ND
34	Gansu05-1-GS-CHN-2005	China	2005	GQ294574	ND
35	JB141230172	China	2012	KC867077	ND
36	JB141230166	China	2012	KC867076	ND
37	JB141230009	China	2012	KC867075	ND
38	JB14080245	China	2008	KC867074	ND
39	N_924	India	2009	JN203530	ND
40	N_828	India	2009	JN203529	ND
41	N_683	India	2008	JN203528	ND
42	N_656	India	2008	JN203527	ND
43	N_452	India	2007	JN203526	ND
44	N_451A_IND07	India	2007	JN203525	ND
45	NIV56210_IND05	India	2005	KF412927	ND
46	A34-YN-CHN-2014	China	2014	LC013414	ND
