# Supplementary material for: Enterovirus Migration Patterns between France and Tunisia
Source: PLoS One. 2015 Dec 28;10(12):e0145674. doi: 10.1371/journal.pone.0145674 (PMC4692522; doi:10.1371/journal.pone.0145674)
Supplement: S3 Table — (DOCX) [file pone.0145674.s006.docx]

**S3 Table. General features of the 1D/VP1 gene sequence datasets used in the study.**

| Enterovirus type | Features | Datasets 1 ^a^  (complete or near-complete gene sequences) | Datasets 2 ^b^  (partial gene sequences, 5’ part) |
| --- | --- | --- | --- |
| E-5 | Number of sequences | 28 | 49 |
|  | Length of sequences (nucleotide positions) | 762 (112 – 873) ^b^ | 276 (160 – 435) |
|  | Geographical locations | 7 | 10 |
| E-9 | Number of sequences | 59 | 184 |
|  | Length of sequences (nucleotide positions) | 732 (34 – 765) ^b^ | 265 (163 – 427) |
|  | Geographical locations | 9 | 21 |
| E-18 | Number of sequences | 64 | 127 |
|  | Length of sequences (nucleotide positions) | 861 * | 357 (142 – 498) |
|  | Geographical locations | 6 | 9 |
| CV-A9 | Number of sequences | 63 | 185 |
|  | Length of sequences (nucleotide positions) | 897 * | 249 (139 – 387) |
|  | Geographical locations | 9 | 24 |

^a^ The asterisks indicate the datasets comprising only complete 1D/VP1 gene sequences. The datasets for the enterovirus types E-5 and E-9 were constructed with partial gene sequence including a major central portion of the 1D/VP1 gene; the nucleotide positions indicated are those of reference sequences (accession numbers AF083069 and AF524866) determined for the E-5 and E-9 prototype strains respectively.

^b^ The alignments were constructed by selecting the largest number of sequences which shared as many nucleotide positions as possible within the 5’ part of the 1D/VP1 gene. The nucleotide positions common to all partial sequences are indicated. The numbering of nucleotide positions refers to the following reference sequences: CV-A9, D00627; E-5, AF083069; E-9, AF524866; and E-18, AF317694.
