# Supplementary material for: Enterovirus Migration Patterns between France and Tunisia
Source: PLoS One. 2015 Dec 28;10(12):e0145674. doi: 10.1371/journal.pone.0145674 (PMC4692522; doi:10.1371/journal.pone.0145674)
Supplement: S4 Table — (DOCX) [file pone.0145674.s007.docx]

| **S4 Table. Spatial diffusion assessed with the phylogenetic data inferred from the 5' partial 1D/VP1 sequences of four enterovirus types and comparison with the spatial diffusion assessed with other sequence datasets.** | | | | | |
| --- | --- | --- | --- | --- | --- |
| **Enterovirus type** | **5' partial sequence dataset** | | | **Complete (or near-complete) dataset** | |
|  | **Countries involved ^a^** | | **Bayes factor ^b^** | **Bayes factor** | **Empirical transportation event** |
| E5 | FRA | TUN | 5460.04 | 183.74 | T1 and T2 |
| E5 | AUS | KOR | 20.98 |  |  |
| E5 | AUS | IND | 12.06 |  |  |
| E9 | KGZ | RUS | 774.99 |  |  |
| E9 | BEL | FRA | 508.67 |  |  |
| E9 | CHN | FIN | 157.85 |  |  |
| E9 | ESP | FRA | 149.187 |  |  |
| E9 | AUS | MYS | 68.16 |  |  |
| E9 | ESP | GBR | 45.55 |  |  |
| E9 | FRA | TUN | 31.63 | 31.02 | T3 and T4 |
| E9 | FRA | NLD | 26.54 |  |  |
| E9 | FRA | ITA | 20.65 |  |  |
| E9 | AUS | FRA | 13.95 | 15.47 | T5 |
| E9 | DNK | FRA | 10.57 |  |  |
| E9 | CHN | FRA | 10.33 |  |  |
| E18 | BEL | FRA | 14132.22 |  |  |
| E18 | AUS | FRA | 138.92 | 489.69 | T6 and T7 |
| E18 | FRA | JPN | 40.62 |  |  |
| E18 | FRA | TUN | 11.46 |  |  |
| CVA9 | FRA | GBR | 95829.79 |  |  |
| CVA9 | CHN | JPN | 3184.03 |  |  |
| CVA9 | CAN | CHN | 2984.36 | 73.89 | T8 |
| CVA9 | GBR | NLD | 478.33 |  |  |
| CVA9 | IND | PAK | 404.24 |  |  |
| CVA9 | AUS | FRA | 262.4 |  |  |
| CVA9 | ITA | TUN | 241.56 |  |  |
| CVA9 | ITA | TWN | 203.75 |  |  |
| CVA9 | GBR | TUN | 137.25 |  |  |
| CVA9 | DNK | FRA | 111.59 |  |  |
| CVA9 | DNK | MOR | 65.17 |  |  |
| CVA9 | FRA | SWE | 55.99 |  |  |
| CVA9 | CHN | THA | 40.14 |  |  |
| CVA9 | CHN | FIN | 33.76 |  |  |
| CVA9 | CHN | FRA | 26.71 | 10.75 |  |
| CVA9 | CHN | TWN | 24.95 |  |  |
| CVA9 | FRA | RUS | 23.33 |  |  |
| CVA9 | FRA | USA | 12.73 |  |  |
| ^a^ Countries involved in the spatial diffusion. The countries are indicated in alphabetical order; this order does not indicate a direction for the virus migration. | | | | | |
| ^b^ The spatial diffusion events assessed with Bayes factor values ranging from 3 to 10 were not indicated. | | | | | |
| Abbreviations used for country designations: AUS, Australia; BEL, Belgium; CAN, Canada; CHN, China; DNK, Denmark; ESP, Spain; FIN, Finland; FRA, France; GBR, Great Britain; IND, India; ITA, Italy; JPN, Japon; KOR, South Korea; KGZ, Kyrgyzstan; MOR, Morocco; MYS, Malaysia; NLD, Netherlands; PAK, Pakistan; RUS, Russia; SWE, Sweden; THA, Thailand; TUN, Tunisia; TWN, Taïwan; USA, United States. | | | | | |
